# Sociovirology: Conflict, Cooperation, and Communication among Viruses

**DOI:** 10.1016/j.chom.2017.09.012

**Published:** 2017-10-11

**Authors:** Samuel L. Díaz-Muñoz, Rafael Sanjuán, Stuart West

**Affiliations:** 1 Department of Microbiology and Molecular Genetics, University of California, Davis, One Shields Avenue, Davis, CA 95616, USA; 2 Institute for Integrative Systems Biology (I2SysBio), Universitat de València, C/Catedrático Agustín Escardino 9, 46980 Paterna Valencia, Spain; 3 Department of Genetics, Universitat de València, C/Dr. Moliner 50, Burjassot, València 46100, Spain; 4 Department of Zoology, University of Oxford, South Parks Road, Oxford OX1 3PS, UK

## Abstract

Viruses are involved in various interactions both within and between infected cells. Social evolution theory offers a conceptual framework for how virus-virus interactions, ranging from conflict to cooperation, have evolved. A critical examination of these interactions could expand our understanding of viruses and be exploited for epidemiological and medical interventions.

## Main Text

**Figure 1:**
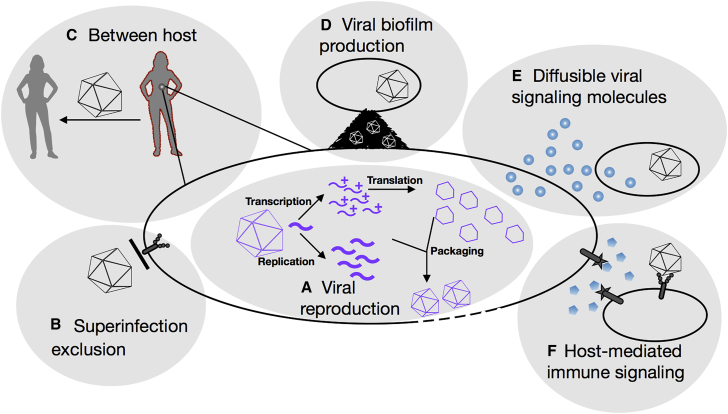
Virus-Virus Interactions Are Diverse and Provide Multiple Opportunities for Social Evolution (A–F) Indicated by gray shadows and depicting social interactions, where the action of a viral genome changes the fitness of other viral genomes. Cells are ovals with black borders. (A) A viral genome (thick curved segment) enters the cell and performs transcription (mRNAs, thin curved segments +) and translation, leading to generation of shared intracellular viral proteins (open hexagons). Transcription-translation is a social and potentially cooperative trait because it benefits the other viral genomes in the cell. (B–F) Viral genomes can prevent or promote reproduction of other genomes by changing the probability that they can infect a cell or host by: (B) blocking the entry of other viral genomes into the cell; (C) producing host-level immune changes (dark red outline) that favor the transmission of all infecting viral genomes; (D) inducing the cell to produce molecules essential for transmission to neighboring cells, benefiting all viral genomes in the cell; (E) producing viral proteins that communicate cell infection status, signaling to other viral genomes the abundance of cells available for reproduction; and (F) manipulating host immune signals to induce distant cells to differentially expose receptors favoring entry of some viral genomes over others.

Virus-virus interactions are pervasive and highly diverse (DaPalma et al., 2010; Figure 1). Some viruses need another, “helper” virus to complete their infection cycle, and other viruses are commonly activated or suppressed by the presence of secondary viral infections. Viral proteins can mix and produce mosaic-like viral particles (pseudotypes) when a cell is coinfected with two different viruses. Viral coinfection of microbes is widespread (Díaz-Muñoz, 2017), and viruses have mechanisms enabling multiple viral genomes to be cotransmitted in the same infectious unit (reviewed in Sanjuán, 2017). Coinfecting viral genomes can be distinct, variants of the same virus, or even genetically identical, suggesting different types of functional interplay. Furthermore, bacteriophages use a form of communication to regulate lysis of the infected cell (Erez et al., 2017). Finally, virus-virus interactions in the absence of cellular coinfection can also be mediated by changes at the host level, such as immune responses.

Despite this growing body of empirical evidence suggesting virus-virus interactions, we lack a well-founded conceptual framework that provides an understanding of how these interactions have evolved and how they could shape viral pathogenesis. Social evolution theory was originally developed to explain animal behavior, but has since been extended to microorganisms, including bacteria and unicellular eukaryotes. Yet this social perspective has not been embraced in the study of viruses.

Potential misgivings with this social-evolution approach include the idea that viruses are too simple to interact socially, or that to infer traits like cooperation would be anthropomorphic. On the flip side, social interactions have driven the evolution of life at all levels of complexity and thus could have a role in viral evolution as well: genes cooperate to form genomes, cells cooperate to form multicellular organisms, and multicellular organisms form complex cooperative societies. Conflict is also ubiquitous: “selfish” genes drive their transmission at a cost to the genome, cells exploit collectively produced goods like nutrient-scavenging molecules, and organisms compete with each other for resources. Since no complex phenotypes are necessary for the evolution of social traits, the appearance of anthropomorphism or the “simple” organization of viruses may not pose problems for the application of sociobiology models in virology.

We argue for a social evolution approach to understand and predict virus-virus interactions. This framework can clarify unexplained phenomena in virus-virus interactions by identifying evolved viral social traits, their genetic and mechanistic basis, and the selective pressures underlying these traits. We outline how this approach could lead to new breakthroughs in both fundamental and applied virology.

### Cooperation and the Individual

**Figure 2:**
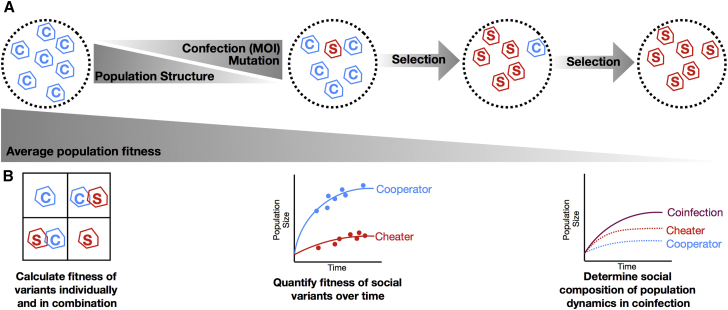
The Problem of Cooperation and Testing Virus-Virus Interactions Using Social Evolution Theory (A) Imagine a population of viruses that perform a costly cooperative action such as transcription (indicated by C). A selfish cheater that does not perform the cooperative action (indicated by S) arises in this population through mutation or coinfection. This selfish cheater is able to benefit from the cooperative behavior of the cooperators without paying the cost. Consequently, the selfish cheater will increase in frequency, even though this leads to a reduction in mean fitness. (B) Testing for evolved social interactions, such as cheating and cooperation. Left: Strains are competed in isolation and in combination to quantify fitness and confirm social traits. Middle: When grown as single infections, a population of cooperators (blue) achieves higher growth than a cheater population (red). Right: In coinfection (purple), the population has lower growth than a population composed solely of cooperators. The social composition of the population reveals why: cheaters (dotted red) initially increase population growth by exploiting cooperators (dotted blue), but growth stalls as the population becomes dominated by cheaters.

Social interactions take place when the traits of one individual influence the fitness of another individual. As such, evolutionary analysis of social interactions relies fundamentally on natural selection. The only major difference with non-social models is that two or more interacting individuals are involved. For example, cooperative traits, which benefit others, will only be selected for by natural selection if they provide a fitness benefit to the individual performing them (Figure 2A). Cooperative traits cannot evolve merely because they provide a benefit to a population. Furthermore, the evolution of cooperation does not require the ability of individuals to foresee the consequences of their actions. In other words, cooperation and social evolution can be explained by, and are not at odds with, individual-based natural selection.

One way cooperation can be evolutionarily favored is when it is directed toward genetically identical individuals or relatives. From an evolutionary perspective, helping a genetically identical individual reproduce is the same as reproducing yourself. By extension, helping relatives reproduce provides a fitness benefit to the actor, as relatives share a fraction of the actor’s genes. This process by which individuals increase their fitness by helping relatives reproduce is termed kin selection.

An important potential difficulty in applying social evolution to virology resides in the definition of an individual. One may consider the virion as an individual. Yet in some cases, including paramyxoviruses, birnaviruses, filoviruses, retroviruses, and inoviruses, a virion can carry multiple genome copies (n-ploidy). Functionally, these n-ploid virions should be similar to multiple virions entering the same cell, because they would both lead to infections with multiple viral genomes. As such, we suggest that the definition of an individual should be set at the level of the single infectious viral genome.

Another potential complication stems from the fact that some viruses, notably RNA viruses, show extremely high mutation rates, leading to the suggestion that the minimal level at which an individual RNA virus can be defined is a sequence cloud, or quasispecies (Andino and Domingo, 2015). In this case, the individual could be redefined as the consensus (or predominant) sequence of the quasispecies. If, however, functional social interactions are established among genetic variants within such clouds, then it remains useful to keep the definition of the individual at the single viral genome level, because this allows identifying and analyzing such interactions.

If we think of a genome as an individual, then the natural history of viruses is filled with opportunities for social interactions (Figure 1), even though experimental demonstration is still lacking in many cases. A single viral genome entering a cell needs to accomplish both replication and gene expression (transcription and translation). The mRNAs and proteins resulting from viral gene expression can provide a collective benefit to genomes in the cell, such as generating capsids and proteins that block host immunity. The fact that these factors act as public goods permits, but does not guarantee, cooperation (Chao and Elena, 2017). Cooperation is more likely to evolve if most interacting genomes are identical, since such high relatedness allows kin selection to operate (Turner and Chao, 1999). Studied with this social evolution lens, even the infection and replication of a single viral genome within a single cell is an inherently social process (Figure 1A).

### Conflict and Cheating Viruses

Evidence in favor of viruses acting as cooperative social agents is, paradoxically, provided by the occasional spread of uncooperative “cheats.” If individuals are not genetically identical, they will not necessarily have the same evolutionary interests, creating the potential for conflict. Social evolution theory predicts that if multiple genomes infect a cell, one that invests less in cooperative traits such as transcription and invests more in its own replication will be favored, because it will benefit from cooperation without paying as much of the cost (Chao and Elena, 2017). This is analogous to the tragedy of the commons in humans, where cooperation breaks down due to selfish interests, even though everyone could do better in the long run by cooperating.

Two important variables determine the extent to which multiple genomes infect a cell: the multiplicity of infection (MOI) and the rate of spontaneous mutation. High mutation rates such as those exhibited by RNA viruses result in extremely diverse populations, whereas a high MOI can bring genomes from different lineages into the same host cells. These variables can enable genomes to interact in coinfection, as long as there is sharing of viral products within cells.

A clear experimental test of the association between MOI and cheater evolution was generated with bacteriophage phi6 (Turner and Chao, 1999). Serial passage at high MOI led to the evolution of a cheater virus optimized to increase its fitness in coinfection by favoring its replication over other coinfecting viruses, reducing average population fitness. In contrast, passage at low MOI selected a virus optimized to grow efficiently in monoinfection and to high population fitness. Thus, coinfection generates conditions ripe for the emergence of cheats.

Defective interfering particles (DIPs) appear to be “cheats.” DIPs usually have a large portion of their genome deleted, including essential proteins, and hence can only reproduce in the presence of completely functional “helper” viruses. However, their smaller genomes provide a replication advantage, outcompeting helper viruses in mixed infections of cells and severely reducing viral population fitness. In the laboratory, artificially high MOIs are well-known to promote the emergence of DIPs (Marriott and Dimmock, 2010). However, a pending challenge is to measure MOIs *in vivo* and to determine the ensuing levels of intra-host genetic relatedness in viruses.

In turn, the association between mutation rate and cheating has been shown in experimental populations of RNA viruses treated with base analog mutagens. This selected for a fraction of viral genomes with DIP-like behavior, greatly reducing population fitness (reviewed in Andino and Domingo, 2015).

Finally, evidence that viral proteins can function as public goods inside the cell is supported by well-known processes in virology, such as pseudotyping, in which a viral particle contains the envelope proteins of a different virus. However, many plus-strand RNA viruses replicate in well-defined subcellular structures. This situation suggests that, in some cases, intracellular viral product sharing might be restricted, which would tend to prevent the spread of cheaters. Thus, the details of viral population structure at the intracellular level and the identification and mechanistic characterization of public goods will be crucial milestones to reach in the sociovirology field.

### Diversity and Heterotypic Coinfection

As detailed above, kin selection theory predicts that high coinfection rates select against cooperation, because they reduce genetic relatedness. However, mutually beneficial cooperation involving genetically distinct individuals (“heterotypic cooperation”) can also evolve. All that is required is that the individuals have some shared interest, such that they have higher fitness if they cooperate.

Cooperation between different genetic variants is a familiar concept in virology, but rigorous evidence supporting it remains scarce. The finding that population genetic diversity correlated with the ability of poliovirus to cause disease in mice led to the suggestion of cooperation, with similar processes postulated for hepatitis B virus, among others (reviewed in Andino and Domingo, 2015). Measuring genetic diversity (e.g., using quasispecies theory or mutation-selection balance) is a first step to establish that interactions potentially exist, but does not provide information on the nature of the interactions and their fitness outcomes.

Genetic complementation, whereby the genetic defects of the interacting viruses are mutually compensated, is not intrinsically cooperation, but represents a possible mechanism for heterotypic cooperation. A potential example comes from influenza viruses, when one strain has a more efficient hemagglutinin, which mediates virus attachment to host cells, and another has a more efficient neuraminidase that facilitates the release of virions from infected cells. In cell culture, these viruses reproduce better together than they do independently, because one is advantaged in cell entry and the other is advantaged in cell exit (Xue et al., 2016). Multiple genome infections also lead to higher viral growth in measles virus and even gain of functions, such as extended cell tropism (Shirogane et al., 2012). A possible reason why interacting genomes may surpass wild-type fitness is that some beneficial mutations show strong negative epistasis when combined in the same viral genome.

Adaptive immunity evasion in hepatitis C virus provides another suggested instance of cooperation. Some variants may encode dominant antigens to monopolize immune responses and favor other viruses, leading to a complex network of cross-immunoreactivity that mitigates the immune pressure acting on certain variants (Skums et al., 2015). As opposed to the above examples, this may not be a mutually beneficial interaction, because the variants encoding the flag antigen would play an altruistic role (Figure 1F). To what extent this outcome is evolutionarily stable is unclear because, as discussed above, altruistic cooperative behavior is critically dependent on genetic relatedness and kin selection.

### Cooperation or Not Cooperation?

As a note of caution, many of the above examples of distinct viral genomes engaging in positive or mutually beneficial interactions are not necessarily cooperation. Whereas cooperation is colloquially used as a synonym for “helping,” in evolutionary terms it requires that a trait provides a benefit to another individual (mutual benefit or altruism) and that the trait has evolved at least partially due to this benefit (West et al., 2007). This latter clause is required because we are interested in whether the benefit to others is an adaptation (Figure 2), and not just a byproduct of an otherwise selfish trait. For example, when an elephant produces dung, this is beneficial to the elephant, but also beneficial to a dung beetle that uses that dung. However, elephants have clearly not evolved defecation to help dung beetles, and so although defecation is mutually beneficial, it is not cooperation.

Thus, the challenge resides in explaining cooperative traits that evolved to benefit others, which becomes difficult with complex interactions. Intercellular virus-virus interactions mediated through changes in the host, such as breakage of physical barriers or immune suppression, could easily fall into the elephant-dung beetle category. Similarly, some of these synergistic interactions reported as heterotypic cooperation between virus variants may be transient.

The designation of byproduct rather than cooperation does not imply that the observed phenomena are not important. Rather, they raise different questions about why they are favored by natural selection, which can be crucial for understanding and manipulating these viral social interactions. The utility of identifying cooperation or cheating, as opposed to a transient interaction, is that they represent evolved social interactions. This identification has two consequences. First, we can uncover the genetic underpinnings of the trait. For instance, the specific mechanism underlying cheating by DIPs has led to DIP-derived treatments that depend only on the gene, rather than the entire viral particle. Second, we can establish the environmental and social pressures that led to selection of that trait. This knowledge of the selective regime can lead to generalizable principles, such as low-MOI vaccine propagation to avoid the appearance of DIPs.

In practice, the key first step will be to test whether evolved social interactions are really occurring between strains, and what form they take (Figure 2B). First, one can measure the fitness of each partner alone and in combination (Bordería et al., 2015). This will show whether a given interaction is beneficial or detrimental, and whether it can be exploited by potential cheats. Second, to show an evolved social interaction, as opposed to a transient interaction, the genetic mechanisms underlying the interaction can be uncovered and exploited in experimental manipulations. For instance, one can engineer strains with and without the trait to test whether a beneficial trait is cooperation or a detrimental trait is cheating. This experimentation can falsify alternative, non-adaptive explanations such as incidental interactions. Finally, if the conditions favoring a cooperative or cheating interaction are known or hypothesized, evolutionary experiments in which these conditions are manipulated can provide further conclusive evidence for an evolved social interaction.

The recently discovered arbitrium system, whereby bacteriophages produce virus-encoded small peptides that are detected by specific virus-encoded receptors in neighboring cells, provides a good test case (Erez et al., 2017). This system could be a cooperative trait, because it specifically senses phage population density and suppresses lysis. Thus, viruses forgo the chance to produce more progeny to prevent the exhaustion of available host cells. If tests, as described above, determine arbitrium is an evolved social trait, this knowledge can be exploited, for instance, to develop phage therapies that disrupt the arbitrium system, achieving better bacterial clearance.

### Virulence and Social Interactions

Virulence can be a social trait. In the simplest scenario, greater pathogen growth will increase transmission, but also increase host mortality (virulence). Consequently, overall viral transmission can be maximized at an intermediate growth rate, where the host is prudently exploited, with the associated virulence an unavoidable consequence. Myxoma virus provides a potential example of prudent exploitation in the field. Myxoma virus causes a lethal disease known as myxomatosis in the European rabbit and was released as a means to control rabbit populations in Australia and France. However, after years of natural infection, the virus evolved toward slightly reduced fatality rates and prolonged host survival times, increasing opportunities for both vector-borne and direct transmission (Kerr et al., 2015).

Virus or host dispersal can also influence the social evolution of virulence. Consider a spatially structured population with limited dispersal of both virus and hosts. In this case, if a viral strain grows quickly, then it can deplete the local supply of susceptible hosts and hence slow transmission of that strain in the long run. Theoretical models predict that such spatially structured populations will select for lower virulence and hence more prudent host exploitation, as first experimentally verified in an insect virus (Boots and Mealor, 2007).

In contrast to the above example, low virulence can also evolve in response to decreased relatedness. When host exploitation relies on intra- and intercellular cooperation between individual genomes, coinfection can select for cheats, leading to the breakdown of cooperation and, consequently, lower parasite growth and reduced virulence (Turner and Chao, 1999).

### Implications

A social perspective on virus evolution can open new research and treatment possibilities. Social thinking can cast viruses in a new light and question fundamental assumptions to advance basic virology. Similarly, by focusing on virus-virus antagonism and alliances, we may be able to uncover new weapons in our own virus-host battle.

The central role of genetic relatedness and kin selection in the evolution of cooperation can help us better understand key processes in virology, such as superinfection exclusion, which makes the cell refractory to entry of other, potentially non-identical genomes, and why some viruses tend to be transmitted as groups (collective infectious units). The social evolution perspective also draws attention to certain long-held assumptions regarding standard virological practices. For example, studies have found that plaque assays may not yield clonal populations (reviewed in Sanjuán, 2017). Social evolution theory can inform which types of viruses are more likely to be affected by this phenomenon and its impact on routine laboratory studies.

The social evolution perspective also opens the door to engineering of new, better viral interventions by deploying our knowledge of social evolution. New attenuated vaccines using DIPs could be generated by using social principles underlying the emergence of cheats (Marriott and Dimmock, 2010). Theory on cheating and how it impacts population fitness could be used in epidemiology to predict the best strategies to limit viral spread. On the other hand, knowledge of kin-selected clonal reproduction can be used to increase viral yield in vaccine production or to increase viral vector gene delivery. Likewise, knowledge of evolved interactions between bacteriophages could lead to improved phage therapy.

In sum, there is growing evidence that virus-virus interactions are pervasive and that viruses have mechanisms to mediate these interactions. We suggest that these traits are best understood and predicted using a social evolution approach. As such, sociovirology holds promise for breakthroughs in fundamental and applied virology.
